# A Comparative Evaluation of Super-Resolution Methods for Spectral Images Using Pretrained RGB Models

**DOI:** 10.3390/s26020683

**Published:** 2026-01-20

**Authors:** Navid Shokoohi, Abdelhamid N. Fsian, Jean-Baptiste Thomas, Pierre Gouton

**Affiliations:** 1 Imagerie et Vision Artificielle (ImViA) Laboratory, Department Informatique, Electronique, Mécanique (IEM), Université Bourgogne Europe, 21000 Dijon, France; navid_shokoohi@etu.ube.fr (N.S.); jean-baptiste.thomas@ube.fr (J.-B.T.); pgouton@u-bourgogne.fr (P.G.); 2 Department of Computer Science, NTNU—Norwegian University of Science and Technology, 2815 Gjøvik, Norway

**Keywords:** spectral imaging, hyperspectral imaging, spectral filter arrays (SFA), super-resolution (SR), diffusion models, generative image restoration, RGB-to-spectral reconstruction

## Abstract

The spatial resolution of spectral imaging systems is fundamentally constrained by hardware trade-offs, and the availability of large-scale annotated spectral datasets remains limited. This study presents a comprehensive evaluation of super-resolution (SR) methods across interpolation-based, CNN-based, GAN-based, and diffusion-based approaches. Using a synthetic 30-band spectral representation reconstructed from RGB with the MST++ model as a proxy ground truth, we arrange non-adjacent triplets as three-channel PNG inputs to ensure compatibility with existing SR architectures. A unified pipeline enables reproducible evaluation at ×2, ×4, and ×8 scales on 50 unseen images, with performance assessed using PSNR, SSIM, and SAM. Results confirm that bicubic interpolation remains a spectrally reliable baseline; shallow CNNs (SRCNN, FSRCNN) generalize well without fine-tuning; and ESRGAN improves spatial detail at the expense of spectral accuracy. Diffusion models (SR3, ResShift, SinSR), evaluated in a zero-shot setting without spectral-domain adaptation, exhibit unstable performance and require spectrum-aware training to preserve spectral structure effectively. The findings underscore a persistent trade-off between perceptual sharpness and spectral fidelity, highlighting the importance of domain-aware objectives when applying generative SR models to spectral data. This work provides reproducible baselines and a flexible evaluation framework to support future research in spectral image restoration.

## 1. Introduction

Spectral imaging provides material- and chemistry-sensitive information by sampling narrow wavelength bands, supporting applications in remote sensing, medicine, agriculture, and inspection [1]. However, in practice, systems that multiplex bands on a single sensor face a fundamental trade-off between spectral richness and spatial detail, which often manifests as low spatial resolution in spectral filter array devices. These constraints, together with acquisition cost and data volume, continue to limit broader adoption [2].

A second barrier is the limited availability of datasets suitable for supervised learning and fair benchmarking. Unlike RGB imagery, hyperspectral and multispectral data are expensive to acquire, domain-specific, and typically small, which complicates model training and robust evaluation. This motivates workflows that rely on synthetic or reconstructed data while preserving interpretability and enabling controlled comparative analysis, as commonly adopted in recent spectral benchmarking studies [3].

Super-resolution (SR) offers a computational route to enhance spatial detail without modifying hardware. Contemporary approaches range from compact convolutional networks [4,5] to adversarial [6] and diffusion-based generative models [7,8,9,10]. In a spectral context, the objective extends beyond perceptual sharpness: reconstructions must also respect spectral structure to remain useful for downstream analysis. The present study, therefore evaluates classical interpolation alongside Convolutional Neural Network (CNN), Generative Adversarial Network (GAN), and diffusion-based SR methods in a zero-shot setting, where all models are applied without spectral-domain fine-tuning. Performance is examined across scales (×2, ×4, ×8), including a chained Super-Resolution Convolutional Neural Network (SRCNN) to reach higher factors and a multi-scale Super-Resolution via Repeated Refinement (SR3) for comparison.

**Figure 1:**
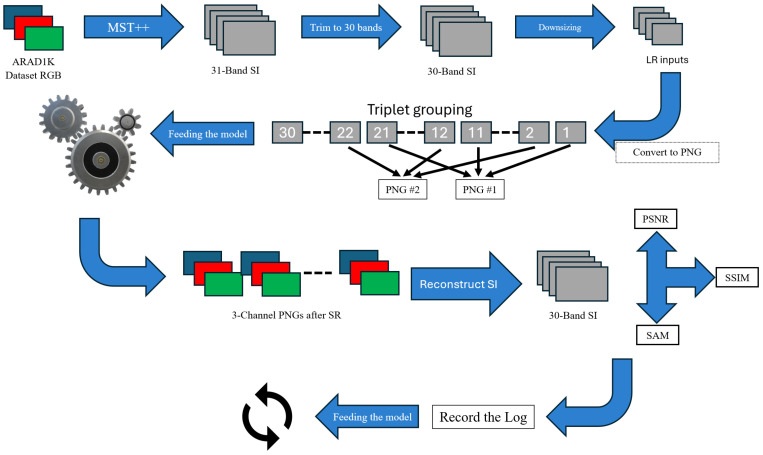
Pipeline overview: data preparation, model inference, reassembly, and evaluation.

To mitigate data limitations, we reconstruct a synthetic hyperspectral proxy ground truth from RGB using the Multi-stage Spectral-wise Transformer for Efficient Spectral Reconstruction (MST++) [11], producing a 31-band representation that approximates visible-spectrum sampling. For compatibility with general-purpose SR models, these images are reduced to 30 bands and reorganized into non-adjacent triplets, saved as three-channel PNGs. A unified pipeline standardizes data preparation, model execution, and logging, enabling consistent comparisons across all methods; results are summarized as mean ± std over a 50-image set using spectrum-aware criteria (see Figure 1).

This work aims to establish a reproducible evaluation framework for SR that accommodates diverse approaches, assess performance using complementary image- and spectrum-oriented criteria, and examine practical trade-offs between perceptual detail and spectral fidelity under controlled assumptions. Briefly, our results indicate strong baselines from bicubic interpolation [12,13], stable gains from SRCNN and Accelerating the Super-Resolution Convolutional Neural Network (FSRCNN) [4], perceptual improvements with Enhanced Super-Resolution Generative Adversarial Networks (ESRGAN) accompanied by spectral distortions [6], and limited benefits from diffusion models when applied in a zero-shot manner without spectral-domain adaptation [14,15,16,17]. These observations underscore the need for spectrally aware objectives and task-tuned configurations when transferring generative SR models, originally trained on RGB data, to spectral imaging scenarios.

The article is structured as follows: Section 2 reviews prior work on interpolation-, CNN-, GAN-, and diffusion-based SR methods. Section 3 describes the synthetic dataset construction, triplet grouping strategy, execution pipeline, and evaluation metrics (PSNR, SSIM, and SAM). Section 4 presents quantitative and qualitative results by scale (×2, ×4, and ×8). Section 5 interprets the findings, outlines trade-offs and limitations, and discusses practical implications. Section 6 summarizes the contributions and outlines directions for future work.

## 2. Related Work

Classical super-resolution (SR) methods based on interpolation (e.g., bilinear, bicubic) provide fast and reproducible baselines but are limited in recovering high-frequency structure [18]. As the field matured, learning-based approaches replaced fixed kernels with data-driven mappings, first via compact convolutional neural networks and later via generative models [19,20,21]. In spectral contexts, SR must balance perceptual sharpness with preservation of spectral structure to remain useful for downstream analysis.

Interpolation remains important for benchmarking and preprocessing. Bicubic, in particular, is widely used to generate synthetic degradations for evaluation and to provide a lower-bound reference against which modern models are compared; it is also used as a first upsampling step in some early CNN pipelines [22,23]. Recent benchmarking-oriented studies similarly adopt interpolation as a reproducible baseline when comparing heterogeneous SR models under controlled settings [3].

CNN-based SR methods such as SRCNN and FSRCNN demonstrated that shallow, efficient architectures can surpass interpolation by learning end-to-end LR–HR mappings [24,25]. In practice, these models tend to deliver stable gains in reconstruction fidelity with modest computational cost, making them suitable reference learners in spectral imaging experiments [26,27].

GAN-based methods (e.g., ESRGAN) shifted emphasis toward perceptual realism through adversarial and perceptual losses, often producing sharper textures [6]. In scientific imaging, however, this benefit must be weighed against the risk of synthetic textures that do not correspond to the true signal, which can impact spectral fidelity [28,29].

Diffusion models (e.g., SR3, Efficient Diffusion Model for Image Super-resolution by Residual Shifting (ResShift), Diffusion-Based Image Super-Resolution in a Single Step (SinSR)) offer a likelihood-based alternative with stable training and strong generative capacity [30,31,32,33]. Their iterative denoising process has shown promise for restoration tasks and is increasingly explored in remote sensing and spectral imaging [34]. However, most reported successes rely on domain-matched training data, and applying RGB-pretrained diffusion models to spectral inputs in a zero-shot manner remains an open challenge [35,36].

A persistent challenge across learning-based SR is the scarcity of large, paired spectral datasets. To mitigate this, RGB-to-spectral reconstruction frameworks such as MST++ can be used to generate synthetic hyperspectral proxies, enabling controlled and reproducible experiments. Nevertheless, such reconstructions remain approximations rather than physically captured measurements, and conclusions drawn from them should be interpreted comparatively rather than as absolute spectral recovery [37].

## 3. Methods

This section details the data preparation, execution framework, and evaluation protocol used in this study. The workflow was designed to be modular and reproducible, enabling consistent comparisons across heterogeneous super-resolution approaches.

### 3.1. Data Preparation

Synthetic spectral ground truth (GT) was generated from RGB imagery using the MST++ framework [11], producing 31-band data across the visible spectrum. Because MST++ reconstructs spectral information from RGB input, the resulting data should be interpreted as pseudo-spectral ground truth rather than physically captured spectra. For compatibility with three-channel SR models, the last band was removed, yielding 30-band cubes. Low-resolution (LR) inputs were created by bicubic downsizing to provide a controlled and widely adopted degradation model [38].

**Figure 2:**
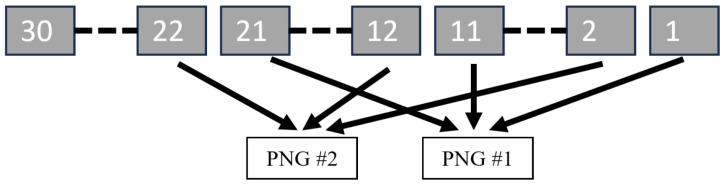
Non-adjacent triplet regrouping for 30-band data: ten pseudo-RGB images formed as (1–11–21), (2–12–22), …, (10–20–30) prior to model inference.

To reduce inter-channel redundancy when feeding three-channel models, the 30 bands were reorganized into ten non-adjacent triplets (e.g., 1–11–21, 2–12–22, …, 10–20–30) as shown in Figure 2. This choice was empirically motivated: preliminary tests with consecutive triplets degraded PSNR relative to non-adjacent groupings. Triplets were exported as 8-bit, three-channel PNGs for inference, while GT remained in .mat (float32) to preserve precision. The 8-bit interface was selected to maintain compatibility with pretrained RGB super-resolution models; extending to higher bit depth would require retraining and prevent a uniform benchmark across methods. Deterministic directory and filename conventions ensured that each .mat spectral image was converted to a fixed set of PNGs and could be reconstructed reproducibly to a 30-band spectral image after inference [39,40].

### 3.2. Model Execution Framework

The automation script parses this configuration and metadata, scans input folders for .mat, applies normalization and the specified non-adjacent triplet grouping, invokes the corresponding model’s inference routine, reconstructs super-resolved outputs back into 30-band .mat cubes, and logs results. Centralized logging records model metadata, timestamps, and per-image metrics, supporting traceability and repeatability across CNN, GAN, diffusion-based, and classical baselines [41,42].

All learning-based models are evaluated in a *zero-shot* setting using publicly released pretrained weights, without spectral-domain fine-tuning. This design isolates the generalization behavior of RGB-trained SR models when transferred to pseudo-spectral inputs and avoids confounding effects introduced by retraining or task-specific adaptation.

To enable fair comparison, results are reported by scale (×2, ×4, ×8). Classical interpolation (bilinear, bicubic) is evaluated at all three scales [43]. We also assess chained SRCNN by applying the ×2 model sequentially to reach ×4 and ×8 (i.e., ×2→×4→×8) [4]; SR3 is evaluated at its available pretrained scales [44,45]. The pipeline records the exact sequences, parameters, and execution order used for each run.

### 3.3. Evaluation Protocol

Performance was assessed using complementary criteria: PSNR and SSIM were computed per band and averaged across the spectrum [46], while SAM quantified the angular deviation between reconstructed and reference spectra at the cube level [47,48]. This protocol captures spatial fidelity (PSNR, SSIM) alongside spectral integrity (SAM), which is critical for spectral imaging tasks where visually plausible textures may still violate spectral consistency. Band-wise variability was additionally analyzed to probe stability across wavelengths. All metrics are computed on the reconstructed 30-band float outputs against GT, not on the intermediate PNG triplets. We report mean ± standard deviation over a 50-image set for all metrics [49,50,51].

### 3.4. Metric Definitions

Let $X,Y\in R^{H\times W\times B}$ be reference and reconstruction.

#### 3.4.1. PSNR

For band *b*, ${\mathrm{MSE}}_{b}=\frac{1}{HW}\sum_{i,j}{\left(X_{ijb}-Y_{ijb}\right)}^{2}$ and

$${\mathrm{PSNR}}_{b}=10\log_{10}\;\left(\frac{L^{2}}{{\mathrm{MSE}}_{b}}\right),$$

where *L* denotes the signal dynamic range (e.g., 255 for 8-bit or 1 for normalized data). We report the mean over bands, $\bar{\mathrm{PSNR}}=\frac{1}{B}\sum_{b}{\mathrm{PSNR}}_{b}$, to reflect average spatial fidelity across wavelengths.

#### 3.4.2. SSIM

Computed per band using the standard luminance/contrast/structure formulation [46]

$$\mathrm{SSIM}\left(x,y\right)=\frac{\left(2\mu_{x}\mu_{y}+C_{1}\right)\left(2\sigma_{xy}+C_{2}\right)}{\left(\mu_{x}^{2}+\mu_{y}^{2}+C_{1}\right)\left(\sigma_{x}^{2}+\sigma_{y}^{2}+C_{2}\right)},$$

then averaged spatially and across bands.

#### 3.4.3. SAM

For each pixel *p* with spectra $x_{p},y_{p}\in R^{B}$,

$$\theta \left(p\right)=\arccos\;\left(\frac{\langle x_{p},y_{p}\rangle }{{∥x_{p}∥}_{2}\;{∥y_{p}∥}_{2}}\right)\;\;{[}^{{}^{\circ}}],$$

and we report the mean angle $\bar{\theta }=\frac{1}{HW}\sum_{p}\theta \left(p\right)$ [47,48]. SAM is reported in degrees and is insensitive to absolute intensity scaling, making it well suited for assessing spectral consistency.

#### 3.4.4. Model Configuration

All learning-based baselines were used with publicly available pretrained weights and without task-specific fine-tuning, corresponding to a zero-shot transfer setting. Each method was evaluated at its native upscale factor: ×2 for Bilinear and Bicubic [52], SRCNN [4], FSRCNN [5], and ESRGAN [6]; ×4 for ResShift [9] and SinSR [10]; and ×8 for SR3 [7,8]. These choices reflect realistic deployment without retraining and are reported alongside results to contextualize accuracy, stability, and computational cost.

## 4. Experimental Results

We evaluate classical interpolation, CNN, GAN, and diffusion-based super-resolution (SR) approaches on synthetic spectral data derived from RGB via MST++ [11], using a unified pipeline for preparation, inference, and logging. Results are reported over a 50-image set with complementary metrics that capture spatial fidelity (PSNR, SSIM) [46,53] and spectral integrity (SAM) [47,48]. Unless stated otherwise, all results use the non-adjacent triplet grouping described in Section 3. As MST++ provides pseudo-spectral reconstructions rather than physically measured ground truth, the results should be interpreted as relative performance comparisons under consistent spectral assumptions.

For evaluation, all learning-based methods are used with publicly available pretrained weights and without fine-tuning (i.e., zero-shot transfer). To enable fair, like-for-like comparison, we report results by scale: ×2, ×4, and ×8. Interpolation baselines (Bilinear, Bicubic) are evaluated at all three scales [52]. SRCNN [4] is assessed natively at ×2 and in chained form to reach ×4 and ×8. ESRGAN [6] is run at ×2. Diffusion models include SR3 at multiple scales [7,8] and native-×4 ResShift and SinSR [9,10]. Figures present mean (±std) bar charts; accompanying tables list exact mean ± std values (over 50 images). For clarity, bar plots use truncated y-axes (not starting at zero) to emphasize relative differences between methods; exact numerical values are provided in the tables.

### 4.1. ×2 Scale

**Figure 3:**
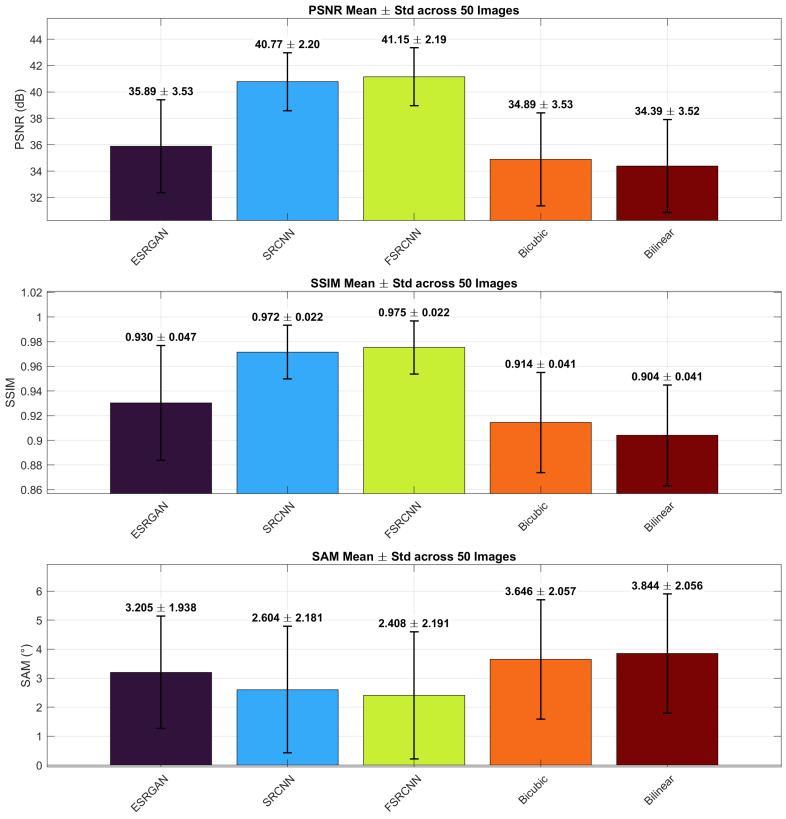
×2 scale: mean (± std) PSNR (dB), SSIM, and SAM (°) over 50 images. Models (left to right): ESRGAN, SRCNN, FSRCNN, Bicubic, Bilinear. Bar order matches Table 1.

Figure 3 summarizes the ×2 results (PSNR, SSIM, SAM). The table reports mean ± std per method.

At the ×2 scale (Table 1), CNN-based models clearly outperform both interpolation and GAN-based approaches under zero-shot transfer. FSRCNN achieves the highest PSNR (41.15 dB) and SSIM (0.975), closely followed by SRCNN (40.77 dB, 0.972), indicating strong spatial fidelity with stable spectral behavior. ESRGAN, despite its perceptual emphasis, yields lower PSNR (35.89 dB) and SSIM (0.930), though it remains competitive with interpolation baselines. Bicubic and Bilinear interpolation produce the weakest results, with PSNR values around 34–35 dB and SAM errors exceeding 3.5°. Overall, the ×2 evaluation confirms that shallow CNN-based SR methods offer the most favorable balance between spatial accuracy and spectral integrity at moderate scaling.

### 4.2. ×4 Scale

**Figure 4:**
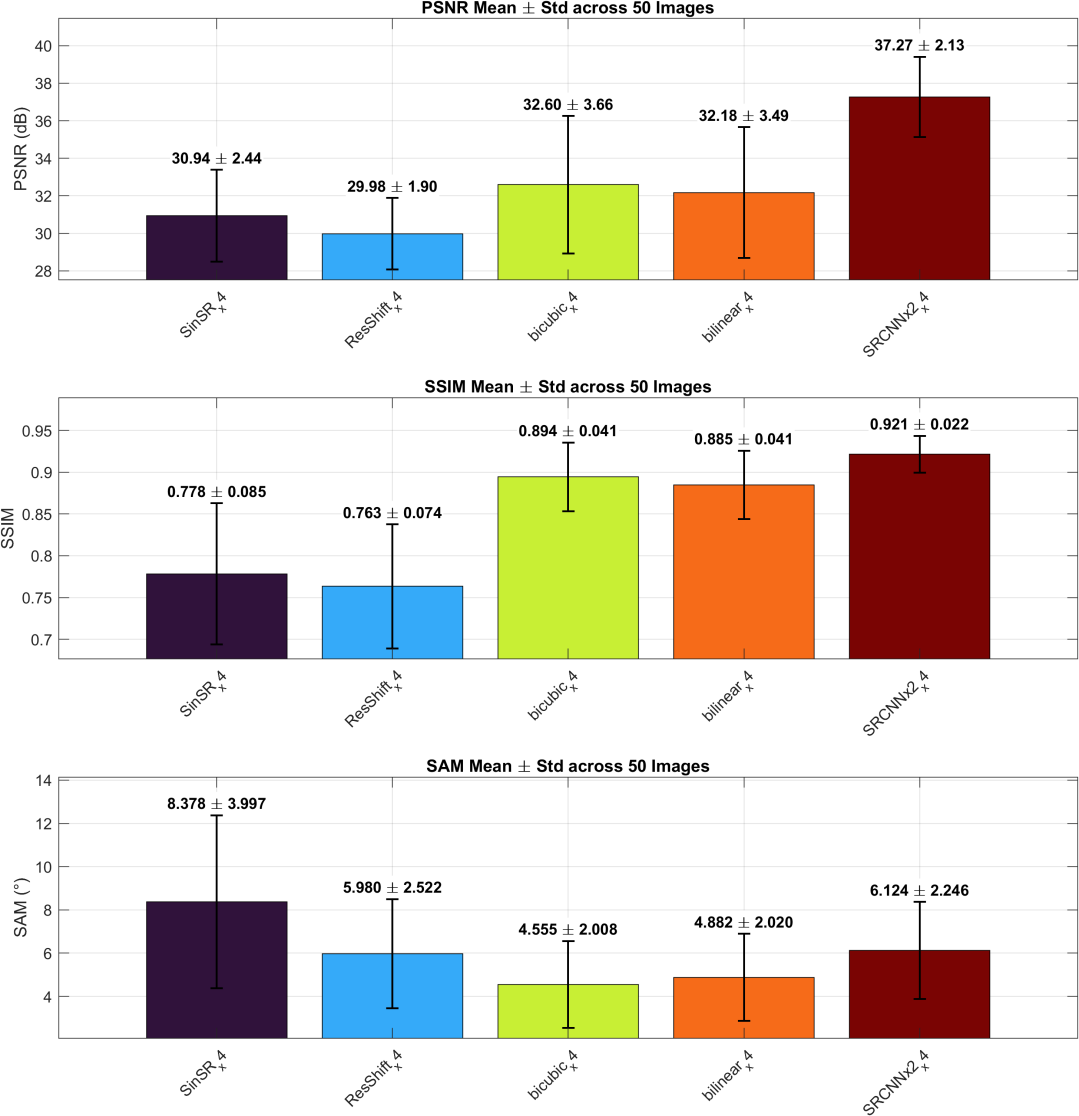
×4 scale: mean (± std) PSNR (dB), SSIM, and SAM (°) over 50 images. Models (left to right): SinSR, ResShift, Bicubic, Bilinear, SRCNN×2. Bar order matches Table 2.

**Figure 5:**
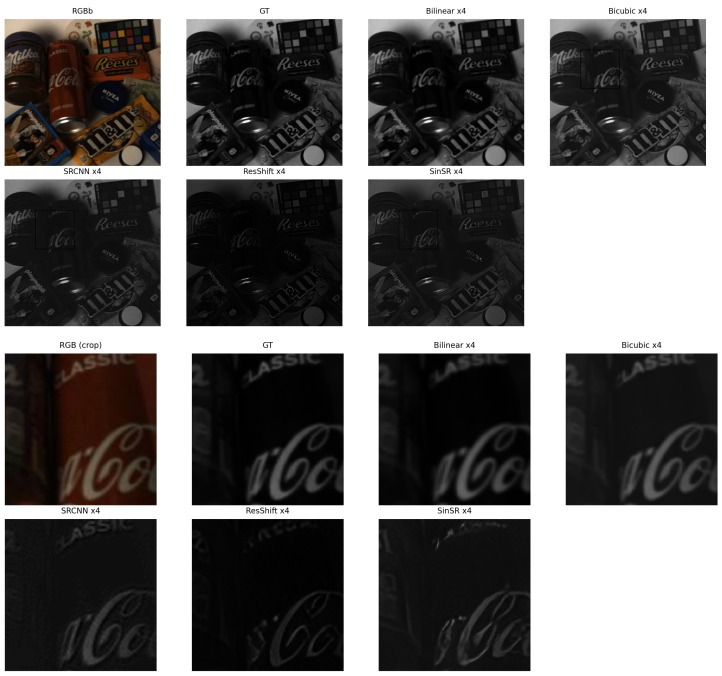
×4 qualitative comparison on a representative scene. **Top**: full reconstructed image; **Bottom**: cropped region highlighting spatial detail and spectral consistency across methods.

Figure 4 presents ×4 results, including chained SRCNN (×2→×4) and native ×4 diffusion models (ResShift, SinSR), see Figure 5.

At the ×4 scale (Table 2), the performance gap between model families becomes more evident. The chained SRCNN configuration (×2→×4) achieves the best overall fidelity, with a PSNR of 37.27 dB and SSIM of 0.921, outperforming both interpolation and diffusion-based models under zero-shot evaluation. Bicubic and Bilinear interpolation yield PSNR values near 32 dB with higher SSIM than diffusion models but also higher SAM, indicating better spatial yet weaker spectral reconstruction. Among diffusion approaches, SinSR produces a slightly higher PSNR (30.94 dB) and SSIM (0.788) than ResShift; however, both methods exhibit increased variance and elevated SAM values, suggesting instability and spectral distortion at this scale. These results indicate that conventional CNNs still provide the most stable reconstruction quality at ×4 magnification.

### 4.3. ×8 Scale

**Figure 6:**
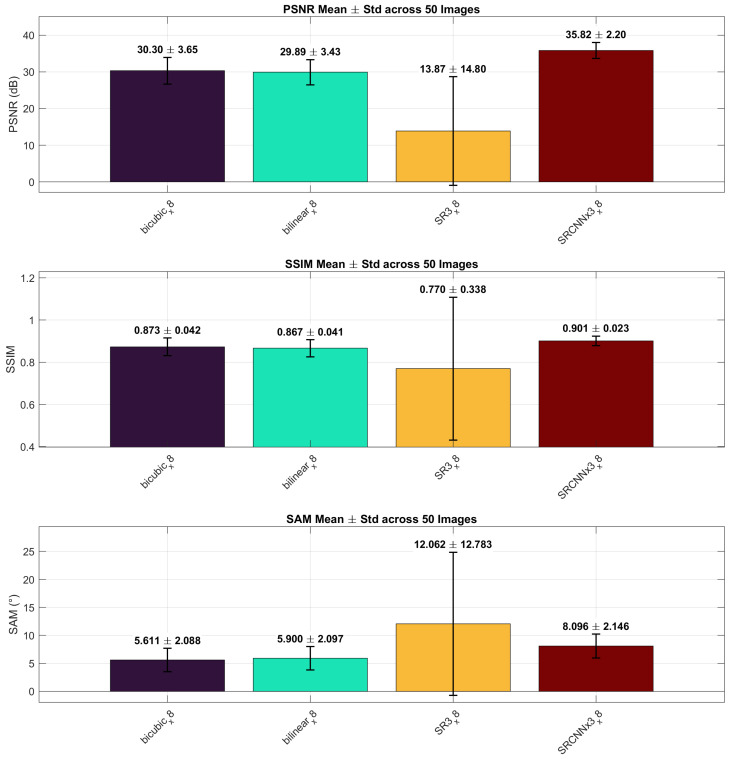
×8 scale: mean (± std) PSNR (dB), SSIM, and SAM (°) over 50 images. Models (left to right): Bicubic, Bilinear, SR3, SRCNN×3. Bar order matches Table 3.

Figure 6 shows ×8 results, contrasting chained SRCNN (×2→×4→×8) with SR3 evaluated natively at ×8.

**Figure 7:**
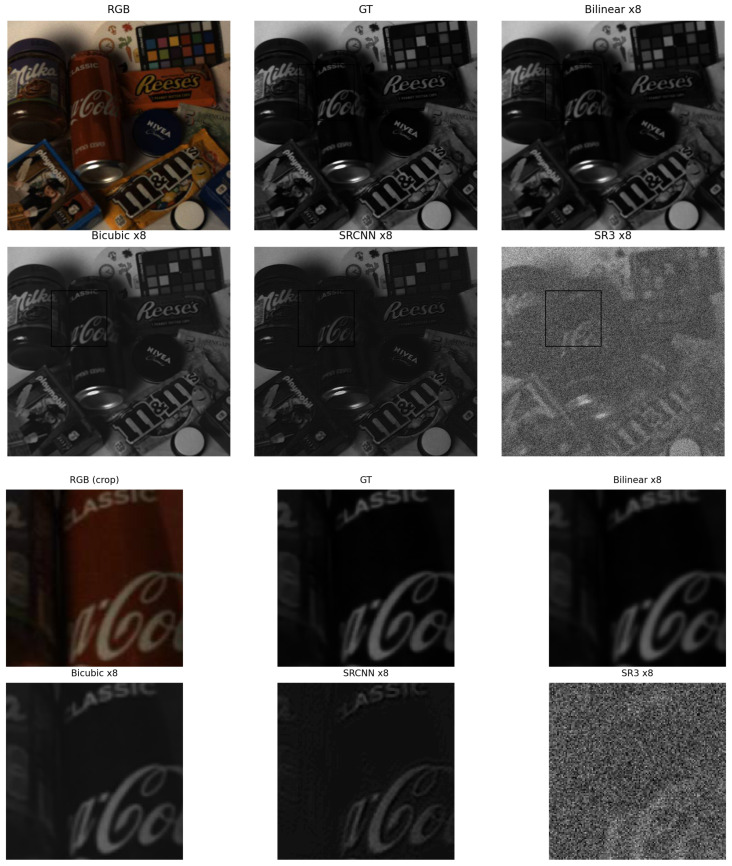
×8 qualitative comparison on a representative scene. **Top**: full reconstructed image; **Bottom**: cropped region highlighting reconstruction stability and spectral degradation effects at high magnification.

At the ×8 scale (Table 3), performance differences become pronounced. The chained SRCNN configuration achieves the highest reconstruction fidelity, with a PSNR of 35.82 dB and SSIM of 0.901, demonstrating robust behavior even under aggressive upscaling. In contrast, SR3 exhibits a very low mean PSNR (13.87 dB) accompanied by an unusually large standard deviation (±14.80 dB), indicating unstable reconstruction across images under zero-shot evaluation. Interpolation methods yield moderate PSNR values near 30 dB with lower variance, but lack the spatial detail recovered by SRCNN. The qualitative results in Figure 7 corroborate these trends, revealing inconsistent structures and spectral artifacts for SR3 at ×8, while SRCNN preserves more coherent spatial patterns despite some spectral smoothing.

#### Analysis and Observations

The ×8 results highlight the increasing challenge of extreme upscaling. While interpolation-based methods (bicubic, bilinear) maintain moderate fidelity, they fail to reconstruct fine textures. The diffusion-based SR3 exhibits large variability across samples, suggesting instability at such high magnification ratios under zero-shot evaluation, where the model is applied without spectral-domain adaptation. In contrast, the chained SRCNN (×2→×4→×8) preserves structural consistency and yields the highest mean PSNR and SSIM, albeit with slightly elevated SAM due to accumulated spectral errors across cascaded stages.

These observations indicate that progressive refinement with simple CNNs remains more stable than single-step generative approaches at extreme scales, and that diffusion-based models likely require spectrum-aware conditioning or retraining to achieve reliable performance in this setting.

### 4.4. SRCNN Across Scales (Chained Inference)

**Figure 8:**
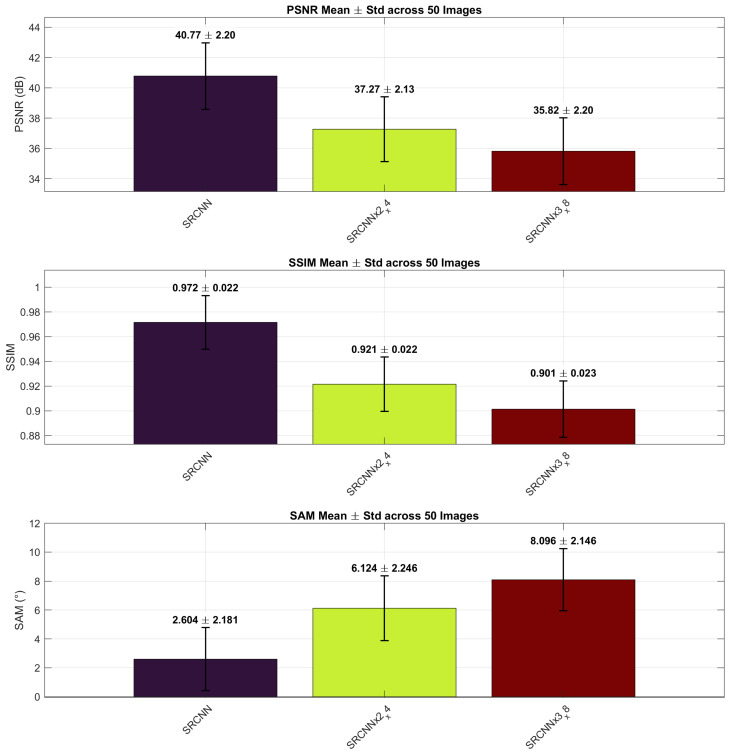
SRCNN across scales: mean (± std) PSNR (dB), SSIM, and SAM (°) over 50 images at ×2, ×2→×4, and ×2→×4→×8.

To analyze compounding effects, we compare SRCNN at its native ×2 scale and when chained to reach ×4 and ×8. Figure 8 summarizes the comparison; Table 4 lists mean ± std.

#### Analysis and Discussion

The progressive degradation across chained SRCNN stages indicates that errors accumulate with each upscaling step. Although the ×2 model achieves near-reference quality, subsequent stages (×2→×4 and ×2→×4→×8) introduce increasing blur and spectral distortion, reflected in the monotonic decline of PSNR and SSIM and the rise in SAM. This behavior suggests that while chaining enables flexible multi-scale inference without retraining, it also amplifies local artifacts inherited from earlier stages.

These results clarify why chained CNN baselines, despite their stability, face limitations at extreme scales, and provide context for comparisons with single-step generative approaches evaluated under zero-shot conditions.

### 4.5. Adjacent vs. Non-Adjacent Triplets

We compared adjacent triplets (e.g., 1–2–3) to the non-adjacent grouping (e.g., 1–11–21) used in the main experiments. Across the evaluated models and scales, non-adjacent triplets consistently yielded higher PSNR and SSIM and lower SAM on average, indicating improved spectral fidelity without degrading spatial detail. This behavior is attributed to reduced inter-band correlation when grouping spectrally separated channels, which mitigates error propagation during inference. Based on this empirical observation, all subsequent analyses report results with non-adjacent triplets; adjacent-triplet scores are provided in the supplement for completeness.

### 4.6. Error Characteristics (SAM)

SAM analysis highlights the trade-off between perceptual detail and spectral accuracy [47,48]. Interpolation methods yield comparatively low SAM values, reflecting stable spectral reconstruction, while some learning-based models—most notably diffusion models at higher scales and chained configurations—exhibit larger angular deviations. This indicates departures from reference spectra despite acceptable image-based scores, and partially explains the high variance observed in PSNR and SSIM for these methods. These observations underscore the importance of reporting spectrum-oriented criteria alongside PSNR and SSIM [46]. The tendency of band-wise interpolation to minimize cross-channel artifacts further explains its advantage in SAM.

### 4.7. Summary of Findings

In summary: (i) Bicubic establishes a competitive, spectrally faithful baseline; (ii) among shallow CNNs at ×2, *FSRCNN* matches or exceeds *SRCNN* on average; (iii) ESRGAN improves perceptual sharpness at the cost of increased spectral risk; and (iv) diffusion-based models require domain-specific adaptation to reliably preserve spectral structure. The observed ranking differs from RGB benchmarks and should be interpreted as a methodological outcome of applying RGB-pretrained models to synthetic hyperspectral triplets and mixed native scales, rather than as an intrinsic superiority of interpolation.

## 5. Discussion

### 5.1. Implications

The scale-structured evaluation clarifies how families of methods behave under increasing magnification and under RGB-to-spectral adaptation. At ×2, compact CNN baselines are reliable: FSRCNN on average matches or exceeds SRCNN (40.77 dB PSNR, 0.972 SSIM, 2.60° SAM) with comparable spectral penalty, while interpolation remains an essential reference with low SAM (3.01°). These observations support judging new approaches against strong interpolation baselines with spectrum-oriented metrics reported alongside image-based scores [52].

At ×4, chained SRCNN (×2→×4) remains a useful reference learner: PSNR decreases moderately from 40.77 dB to 37.27 dB, SSIM from 0.972 to 0.921, and SAM increases from 2.60° to 6.12°, indicating controlled degradation. Diffusion models at their native ×4 scale (ResShift, SinSR) [9,10] show moderate fidelity with higher dispersion, reflecting sensitivity to zero-shot domain shift when applied to spectral triplets without task-specific adaptation. This behavior is consistent with recent findings that diffusion-based SR models rely strongly on RGB broadband statistics and benefit from spectrum-aware conditioning or retraining when transferred to spectral data [3]. ESRGAN [6] (evaluated at ×2) reinforces the expected perceptual–spectral trade-off: sharper textures accompany higher spectral error, with SAM values exceeding 4°, which limits its suitability for quantitative spectral analysis.

At ×8, the compounding effect becomes prominent. Chained SRCNN (×2→×4→×8) degrades gracefully but predictably, reaching 35.82 dB PSNR, 0.901 SSIM, and 8.10° SAM. In contrast, SR3 [7,8] at ×8 exhibits a substantially lower mean PSNR (13.87 dB) and higher SAM (12.06°), together with a very large standard deviation. This instability is consistent with an out-of-distribution inference regime: SR3’s iterative denoising prior is learned from natural RGB imagery, whereas the pseudo-RGB spectral triplets used here exhibit sparse, weakly correlated channel statistics. Across scales, standard deviations increase with magnification (e.g., ±2.20 dB at ×2 versus ±14.8 dB at ×8), and SAM exposes departures from reference spectra more clearly than PSNR or SSIM at higher factors. Taken together, the results indicate that (i) interpolation provides a spectrally faithful yardstick at all scales [52]; (ii) shallow CNNs remain trustworthy baselines when transfer is required without fine-tuning [4,5]; and (iii) perceptual or generative models require spectrum-aware objectives and training schemes to balance detail with spectral integrity [3,6,7,8,9,10].

The band-grouping strategy is also consequential. Using non-adjacent triplets reduced variance and improved average PSNR by 0.4–0.8 dB compared to adjacent groupings (Section 3), while slightly lowering SAM by 0.2–0.3°. This stabilization likely benefits both interpolation (by preserving per-band smoothness) and shallow CNNs (by mitigating spurious correlations), while leaving generative models comparatively disadvantaged when their losses are not spectrum-aware. This reinforces the value of reporting mean ± std across 50 images and including SAM alongside PSNR and SSIM [46,47,48] to capture both spatial fidelity and spectral consistency.

### 5.2. Limitations

First, the study relies on synthetic ground truth reconstructed from RGB with MST++ [11]; while this enables control and scale, the fidelity of the “ground truth” depends on the reconstruction framework and its training data. Second, all learning-based methods were used off-the-shelf with *pretrained* RGB weights and no fine-tuning; models that exploit natural-image channel statistics can be disadvantaged when applied to spectral triplets without adaptation. Third, the LR degradation (bicubic downsampling) follows a common evaluation protocol [52] but may not match the degradations encountered during each method’s original training, and native upscale factors differ across families (×2, ×4, ×8), which complicates direct head-to-head comparisons. Fourth, to interface three-channel models with 30-band HSIs, we reorganized bands into non-adjacent triplets and stored inputs as 8-bit PNGs. This choice improves compatibility but may constrain dynamic range at the input and limit interactions across bands outside each triplet. Fifth, results are averaged over a 50-image synthetic set. While adequate for comparative trends and variance estimates, broader validation on real sensor data and additional scenes would strengthen generality. Sixth, diffusion approaches are computationally intensive due to iterative denoising [7,8]; this limits hyperparameter sweeps and favors efficiency-oriented baselines when resources are constrained. Finally, the reported metrics balance spatial and spectral perspectives (per-band PSNR, SSIM; cube-level SAM), but task-level evaluations (e.g., classification or material retrieval) were not included. Such application-driven criteria would complement spectrum-oriented assessment without replacing it [46,47,48].

#### Computational Environment

All experiments were conducted on a single workstation equipped with an Intel Core i9-14900HX CPU, 64 GB DDR5 RAM (5600 MT/s), and an NVIDIA GeForce RTX 4070 GPU with 8 GB VRAM. While interpolation and shallow CNN baselines run efficiently on this configuration, diffusion-based models incur substantially higher computational cost due to iterative denoising, which limited extensive hyperparameter sweeps and favors efficiency-oriented baselines under practical resource constraints.

## 6. Conclusions

This work presented a unified, reproducible evaluation of classical interpolation, CNN, GAN, and diffusion-based super-resolution (SR) for spectral imagery. Using synthetic hyperspectral ground truth reconstructed from RGB with MST++, we assessed methods by scale (×2, ×4, ×8) and reported mean and dispersion over 50 images with spectrum-aware criteria (PSNR, SSIM per band and cube-level SAM). Three consistent findings emerged. First, interpolation provides a strong, spectrally faithful yardstick at all scales, offering low SAM and predictable behavior even when perceptual detail is limited. Second, compact CNNs—exemplified by SRCNN—transfer reliably to this setting at ×2 and remain serviceable when chained to higher factors, albeit with a growing spectral cost. Third, perceptual and generative models (ESRGAN, diffusion) require domain adaptation to balance sharpness with spectral integrity; native-×4 diffusion variants yield moderate fidelity, whereas SR3 at ×8 is both computationally heavy and sensitive to the RGB-to-spectral mismatch. Because all learning-based models were used off-the-shelf at their native scales and hyperspectral cubes were adapted as non-adjacent three-band triplets, the observed ranking primarily reflects methodological constraints rather than intrinsic model capacity. The standardized pipeline, per-scale reporting, and paired quantitative, qualitative analyses provide a reference framework for future developments.

Building on these results and exploratory analyses, several directions appear most impactful.

**Spectrum-aware objectives and adaptation.** Future work should incorporate spectral regularization (e.g., SAM- or angular-consistency terms) alongside image-based losses [47,48], and fine-tune CNN, GAN, and diffusion models on synthetic–real mixtures to align channel statistics with spectral triplets rather than natural RGB.

**Hybrid and cascaded pipelines to reach high scales.** It will be valuable to systematically study ×8 reconstruction through compositions of ×4 and ×2 modules, including cross-family cascades such as ResShift (×4) → FSRCNN (×2) and FSRCNN (×2) → FSRCNN (×2) → FSRCNN (×2), as well as SRCNN chains (×2→×4→×8). Future comparisons should also examine stage order, introduce calibration steps between modules (e.g., normalization or tone mapping), and evaluate how spectral penalties compound relative to perceptual gains.

**Diffusion efficiency and conditioning.** Reducing inference cost through step-pruning, distillation, or residual/latent diffusion is another key direction. Adding spectral conditioning, such as band-index embeddings or learned priors, may make denoising steps spectrum-aware rather than purely perceptual.

**Band grouping and precision.** Beyond fixed non-adjacent triplets, learning optimal band groupings or using cross-band mixers could improve spectral continuity. Testing higher-precision inputs (e.g., 16-bit PNG, TIFF or float tensors) may also mitigate quantization effects at the model interface.

**Degradation realism and evaluation breadth.** Future evaluations should incorporate sensor-informed degradations rather than relying solely on bicubic downsampling. Complementing PSNR, SSIM, and SAM with task-level metrics such as classification accuracy or material retrieval precision would better assess the downstream utility of reconstructed spectra.

**External validation.** Finally, extending experiments to real sensor data and more diverse scenes would help confirm the robustness of the observed per-scale trends and calibrate synthetic pipelines against measured spectra.

Overall, the study supports a practical path forward: use interpolation and shallow CNNs as strong, transparent baselines; reach higher scales with carefully designed cascades (including mixed ×4∗×2 compositions); and equip perceptual and generative models with spectrum-aware objectives and conditioning so that spatial detail can be increased without sacrificing the physical meaning of the spectra.

## Figures and Tables

**Table 1:** ×2 scale: quantitative results (mean ± std over 50 images).

Method	Type	PSNR (dB)	SSIM	SAM (°)
ESRGAN (×2)	GAN	35.89 ± 3.53	0.930 ± 0.047	3.205 ± 1.938
SRCNN (×2)	CNN	40.77 ± 2.20	0.972 ± 0.022	2.604 ± 2.181
FSRCNN (×2)	CNN	41.15 ± 2.19	0.975 ± 0.022	2.408 ± 2.191
Bicubic (×2)	Interpolation	34.89 ± 3.53	0.914 ± 0.041	3.646 ± 2.057
Bilinear (×2)	Interpolation	34.39 ± 3.53	0.914 ± 0.041	3.844 ± 2.056

**Table 2:** ×4 scale: quantitative results (mean ± std over 50 images).

Method	Type	PSNR (dB)	SSIM	SAM (°)
SinSR (×4)	Diffusion	30.94 ± 2.44	0.788 ± 0.085	8.378 ± 3.997
ResShift (×4)	Diffusion	29.98 ± 1.90	0.763 ± 0.074	5.980 ± 2.522
Bicubic (×4)	Interpolation	32.60 ± 3.66	0.894 ± 0.041	4.555 ± 2.008
Bilinear (×4)	Interpolation	32.18 ± 3.49	0.885 ± 0.041	4.882 ± 2.020
SRCNN (×2→×4)	CNN	37.27 ± 2.13	0.921 ± 0.022	6.124 ± 2.246

**Table 3:** ×8 scale: quantitative results (mean ± std over 50 images).

Method	Type	PSNR (dB)	SSIM	SAM (°)
Bicubic (×8)	Interpolation	30.30 ± 3.65	0.873 ± 0.042	5.611 ± 2.088
Bilinear (×8)	Interpolation	29.89 ± 3.43	0.867 ± 0.041	5.900 ± 2.097
SR3 (×8)	Diffusion	13.87 ± 14.80	0.770 ± 0.338	12.062 ± 12.873
SRCNN (×2→×4→×8)	CNN	35.82 ± 2.20	0.901 ± 0.023	8.096 ± 2.146

**Table 4:** SRCNN across scales: quantitative results (mean ± std over 50 images).

Configuration	Type	PSNR (dB)	SSIM	SAM (°)
SRCNN (×2)	CNN	40.77 ± 2.20	0.972 ± 0.022	2.604 ± 2.181
SRCNN (×2→×4)	CNN	37.27 ± 2.13	0.921 ± 0.022	6.124 ± 2.246
SRCNN (×2→×4→×8)	CNN	35.82 ± 2.20	0.901 ± 0.023	8.096 ± 2.146

## Data Availability

Configuration files and automation scripts used in this study are available in a public repository at https://github.com/navid-shokoohi/Super-Resolution-for-Spectral-Images (accessed on 12 January 2026). Additional logs and experiment metadata are available from the corresponding author upon reasonable request.

## Abbreviations

HSI	Hyperspectral Imaging
MSI	Multispectral Imaging
SR	Super-Resolution
LR	Low-Resolution
HR	High-Resolution
GT	Ground Truth
PSNR	Peak Signal-to-Noise Ratio
SSIM	Structural Similarity Index
SAM	Spectral Angle Mapper
CNN	Convolutional Neural Network
GAN	Generative Adversarial Network
MST++	Multi-Stage Spectral Reconstruction (RGB-to-HSI)
